# Cannabinoid-mediated modulation of neuropathic pain and microglial accumulation in a model of murine type I diabetic peripheral neuropathic pain

**DOI:** 10.1186/1744-8069-6-16

**Published:** 2010-03-17

**Authors:** Cory C Toth, Nicole M Jedrzejewski, Connie L Ellis, William H Frey

**Affiliations:** 1 Department of Clinical Neurosciences and the Hotchkiss Brain Institute, University of Calgary, Calgary, Alberta, Canada; 2 Alzheimer's Research Center at Regions Hospital, HealthPartners Research Foundation, St. Paul, Minnesota, USA

## Abstract

**Background:**

Despite the frequency of diabetes mellitus and its relationship to diabetic peripheral neuropathy (DPN) and neuropathic pain (NeP), our understanding of underlying mechanisms leading to chronic pain in diabetes remains poor. Recent evidence has demonstated a prominent role of microglial cells in neuropathic pain states. One potential therapeutic option gaining clinical acceptance is the cannabinoids, for which cannabinoid receptors (CB) are expressed on neurons and microglia. We studied the accumulation and activation of spinal and thalamic microglia in streptozotocin (STZ)-diabetic CD1 mice and the impact of cannabinoid receptor agonism/antagonism during the development of a chronic NeP state. We provided either intranasal or intraperitoneal cannabinoid agonists/antagonists at multiple doses both at the initiation of diabetes as well as after establishment of diabetes and its related NeP state.

**Results:**

Tactile allodynia and thermal hypersensitivity were observed over 8 months in diabetic mice without intervention. Microglial density increases were seen in the dorsal spinal cord and in thalamic nuclei and were accompanied by elevation of phosphorylated p38 MAPK, a marker of microglial activation. When initiated coincidentally with diabetes, moderate-high doses of intranasal cannabidiol (cannaboid receptor 2 agonist) and intraperitoneal cannabidiol attenuated the development of an NeP state, even after their discontinuation and without modification of the diabetic state. Cannabidiol was also associated with restriction in elevation of microglial density in the dorsal spinal cord and elevation in phosphorylated p38 MAPK. When initiated in an established DPN NeP state, both CB1 and CB2 agonists demonstrated an antinociceptive effect until their discontinuation. There were no pronociceptive effects demonstated for either CB1 or CB2 antagonists.

**Conclusions:**

The prevention of microglial accumulation and activation in the dorsal spinal cord was associated with limited development of a neuropathic pain state. Cannabinoids demonstrated antinociceptive effects in this mouse model of DPN. These results suggest that such interventions may also benefit humans with DPN, and their early introduction may also modify the development of the NeP state.

## Background

Due to its recent epidemic, diabetes mellitus has become the most common cause of peripheral neuropathy worldwide, and approximately 50% of these patients experience chronic neuropathic pain (NeP) [1]. The mechanisms leading to NeP in diabetic peripheral neuropathy (DPN) are likely multifold, but remain poorly understood. As well, management of NeP in general, including that associated with DPN, is inadequate and unsatisfactory [2]. Of the current pharmacotherapies for neuropathic pain, most are designed to block neurotransmission. This may limit their effectiveness as the concomitant production of many inflammatory mediators continues to activate nociceptive neurons, contributing to pain hypersensitivity. It has been demonstrated that injuries and diseases of the nervous system resulting in NeP promote the presence of inflammatory mediators within the spinal cord. Pro-inflammatory cytokines such as interleukin-1beta (IL-1β), interleukin-6 (IL-6), and tumor necrosis factor-alpha (TNFα) are mainly produced by non-neuronal cells, such as with glial cells in the spinal cord, [3,4] and play an unchecked role in the creation of a neuropathic pain state.

The knowledge of the prominent role of glial cells in the development and maintenance of NeP has evolved over the past decade. Both microglia and astrocyte activation is observed in the spinal cord following either peripheral nervous system (PNS) or central nervous system (CNS) injury [5-7]. Glial activation has also been demonstrated in post-traumatic models, inflammatory models [8-10], central demyelinating disorders [11,12], and in diabetes mellitus [13-15]. Escalating evidence suggests that glial cells in the spinal cord play an important role in facilitation of pain [16,17], associated with profound morphological changes in microglia [18].

Furthermore, glial inhibitors or glia modifying drugs such as fluorocitrate and propentofylline can modify pain sensitivity [19,20]. Microglia are regarded as a main source of such inflammatory mediators such as IL-1β, IL-6, and TNFα in the central nervous system [21,22]. Gene regulation following peripheral nervous system injury is dramatically altered in spinal microglia [23], who are also subject to proliferation [24]. Microglia also express a number of plasma membrane receptors whose activation ledas to microglial cell activation [4,25] and migration [4,25]. However, the details of signaling molecules triggerring microglial cell activation and migration are poorly understood.

One important microglial system is the family of cannabinoid (CB) receptors and its endogenous ligands. Endocannabinoids modulate microglial cell migration without disturbing their ability to phagocytose particles or produce nitric oxide. Although endocannabinoids, including anandamide and 2-arachidonoylglycerol (2-AG) [26], act upon CB1 and CB2 receptors are secreted by neurons, they are more prominently produced in microglial cells during neuroinflammatory conditions. 2-AG promotes recruitment of microglial cells by ligation of CB2, but not CB1 receptors [26]. This is likely due to CB1 receptors being expressed predominately at neurons in the central nervous system [27-29], while the CB2 receptor is expressed predominately by immune cells such as microglia [30]. Although both CB1 and CB2 receptors are expressed in activated microglial cells, their cellular expression is different, with CB2 receptors abundantly expressed at the leading edges of activated microglia [26].

Tetrahydrocannabinol (THC), a component in marijuana, acts at both CB1 and CB2 receptors, but other forms of cannabinoids such as cannabinol and cannabidiol act predominantly at CB2 receptors. Such CB2 agonists may be potential anti-inflammatory therapies [31], antagonizing the 2-AG-induced recruitment of microglia and impacting upon development of an inflammatory state. Such properties may permit the cannabinoids to act in the prevention of microglial activation, perhaps limiting the development of neuropathic pain.

We examined clinically available pharmacotherapies for neuropathic pain to identify impact upon microglial activation in a model of chronic neuropathic pain. The aims of our study were to identify the activation of microglia in mice with diabetes and confirmed diabetic peripheral neuropathy. Next, we examined whether CB2 receptor ligation could prevent microglial activation and migration. Finally, we assessed CB1 and CB2 receptor agonists/antagonists in the management of pain and microglial activation in diabetic mice with long standing diabetic neuropathy. We also sought to compare intranasal delivery to intraperitoneal delivery of CB1 and CB2 receptor antagonists since targeted intranasal direct delivery to the CNS may be more beneficial in pain management. Our hypotheses were: (1) microglia activation occurs in spinal cords and thalami of mice with diabetes and painful diabetic peripheral neuropathy; (2) CB2 receptor agonists would prevent microglial activation, ameliorating the development of neuropathic pain; (3) CB1 agonists would benefit pre-existing neuropathic pain in animals with long standing diabetic peripheral neuropathy and associated pain. Our final goal was to determine if nabilone, a synthetic commercially available cannabinoid with CB1 and CB2 agonism activity was beneficial in the diabetic peripheral neuropathy state and could prevent the development of neuropathic pain in this chronic disease state.

In order to specifically target the central nervous system for activation of microglia, cannabinoid agents were intranasally delivered to the brain. Intranasal delivery was first developed to bypass the blood-brain-barrier and directly target growth factors and other therapeutic agents to the central nervous system [32] of rodents [33-35] and humans [34,35] with delivery occurring along both the olfactory and trigeminal neural pathways using extracellular pathways rather than axonal transport [34]. Previous studies have demonstrated that intranasal delivery of cannabinoids achieves both central nervous system and systemic concentrations comparable with systemic delivery [33].

## Methods

### Animals

All experiments were carried out using male CD1 mice (Charles River Laboratories), weighing 25-30 g at initation. All protocols were reviewed and approved by the University of Calgary Animal Care Committee using the Canadian Council of Animal Care guidelines. Experimental study groups were randomized and behavioural studies were performed by an experimenter who was unaware of treatment groups.

In all cases, we studied CD1 male wildtype mice with initial weight of 20-30 g housed in plastic sawdust covered, pathogen-free cages with a normal light-dark cycle and free access to mouse chow and water. CD1 mice were selected due to lower rates of anticipated mortality in long-term diabetic studies [36,37]. Mouse numbers were selected to anticipate 25% and 40% mortality over the course of 6 and 8 months of diabetes respectively. Mice were anesthetized with pentobarbital (60 mg/kg) prior to all terminal endpoints. At the age of 1 month, mice intended to be diabetic were injected with streptozotocin (Sigma, St. Louis, MO) intraperitoneally once daily for each of three consecutive days with doses of 60 mg/kg, 50 mg/kg, and then 40 mg/kg, while mice intended to be non-diabetic were injected with volume-matched carrier (sodium citrate) for three consecutive days.

In all cases, whole blood glucose measurements were performed monthly with puncture of the tail vein and a blood glucometer (OneTouch Ultra Meter, LifeScan Canada, Burnaby, BC, Canada). Hyperglycemia was verified 1 week after STZ injections, with a fasting whole-blood glucose level of ≥ 16 mmol/l (normal 5-8 mmol/l) being our definition for experimental diabetes. Mice that did not meet these criteria for diagnosis of diabetes were excluded from further assessment. All animals were weighed monthly.

### Intranasal and Systemic Delivery of Cannabinoid Agents

Intranasal delivery of the cannabinoid agents took place over the indicated time points for each experiment. While each mouse was held in supine position while in neck extension, a total of 24 μl corresponding to doses of each agent or 0.9% saline only was provided as four drops of 6 μl each through an Eppendorf pipetter over alternating nares every 1 minute. Intraperitoneal delivery of either cannabinoid agents or 0.9% saline only was also administered daily to either diabetic or non-diabetic male CD1 mice as indicated with rotating sites used over the abdomen for injections. Based upon our prior experience with pharmacological delivery to the nervous system [37,38], intranasal dosing was generally 1/10 that of intraperitoneal dosing, which was selected based upon published reports. We chose to use the low dose intervention group as a control group for comparison to higher doses of intranasal or intraperitoneal delivered cannabinoid agents.

In order to determine appropriate infiltration of delivered cannabinoids to the nervous system, FITC fluorescence conjugation was performed (FluoroTag™ FITC Conjugation Kit, Sigma-Aldrich Canada) and fluorescent tagged molecules of cannabidiol and nabilone were delivered through intranasal and intraperitoneal delivery in 4 diabetic mice each, with surgical harvesting of brain and spinal cord tissues of interest at 1, 6, and 24 hours. An additional group of diabetic mice received unlabelled saline both intranasally (n = 4) and intraperitoneally (n = 4) for comparison.

### Study Timelines, Animal Groups and Drug Administration

**Figure 1:**
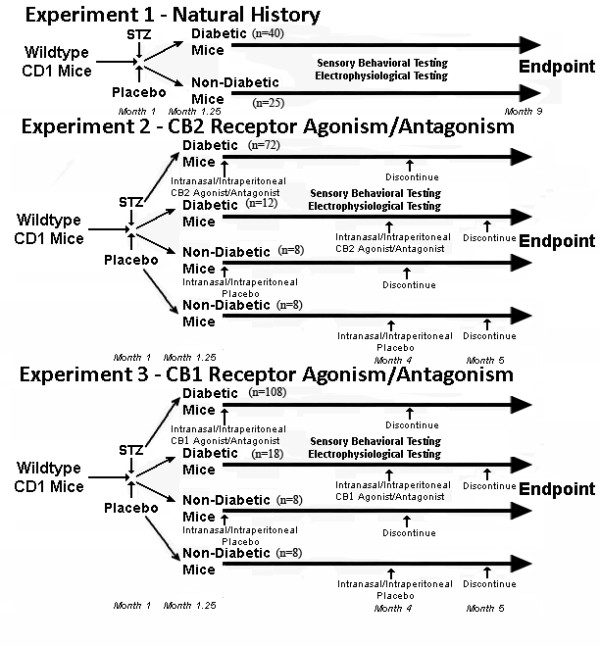
**Timelines for experiments performed**. Experiment 1 examined the natural history of nociception and microglial presence over 8 months of diabetes. Experiment 2 examined the intranasal and intraperitonel delivery of CB2 agonists/antagonists either at diabetes initiation or after diabetic peripheral neuropathy-mediated neuropathic pain has initiated. Experiment 3 examined the intranasal and intraperitonel delivery of CB1 agonists/antagonists (as well as one CB1 and CB2 agonist) either at diabetes initiation or after diabetic peripheral neuropathy-mediated neuropathic pain has initiated.

In Experiment 1, we first sought to study CD1 mice with long term diabetes with comparison to non-diabetic littermates to determine expected levels of tactile allodynia and thermal hyperalgesia as well as expected levels of central nervous system microglial activation (Figure 1). A total of 40 male CD1 wildtype mice were induced diabetic while 25 male CD1 wildtype mice were studied as citrate-injected control littermates over the course of 8 months of diabetes (9 months of life) without delivery of any other agents. These mice were studied with bimonthly sensory behavioral testing, and with monthly sensory nerve conduction studies. Their spinal cords were harvested after 1, 3, 5, and 8 months of diabetes for immunohistochemical and molecular investigations.

In Experiment 2, we examined the impact of CB2 receptor activation or blockade upon microglial densities in the central nervous system in the presence of painful diabetic peripheral neuropathy. Life span in this experiment was chosen to reflect the onset and duration of neuropathic pain identified in Experiment 1. A total of 96 male CD1 wildtype mice were induced diabetic while 16 male CD1 wildtype mice were studied as citrate-injected control littermates over the course of 4.5 months of diabetes (5.5 months of life). A total of 18 diabetic mice received intranasal cannabidiol (selective CB2 agonist) (Cayman Chemical) [39] (n = 6, 0.1 mg/kg; n = 6, 1 mg/kg; n = 6, 2 mg/kg) beginning at one week after STZ injection when diabetes was confirmed with this therapy continued for 3 months. Meanwhile, 18 diabetic mice received intranasal SR144528 (Aztra-Zeneca), a selective CB2 receptor antagonist (n = 6, 0.01 mg/kg; n = 6, 0.1 mg/kg; n = 6, 0.2 mg/kg), again starting at the time of confirmation of diabetes at 1 week post-STZ injections for the duration of 3 months. In order to examine the effect of CB2 receptor agonism in the presence of an established chronic neuropathic pain state, an additional 6 diabetic mice received intranasal cannabidiol (2 mg/kg) and 6 diabetic mice received intranasal SR144528 (0.2 mg/kg) beginning at 4 months of age (3 months of diabetes) for one month. Intraperitoneal delivery was performed for comparison of effect of intranasal delivery. A total of 18 diabetic mice received intraperitoneal cannabidiol (selective CB2 agonist) (n = 6, 1 mg/kg; n = 6, 10 mg/kg; n = 6, 20 mg/kg) and a total of 18 diabetic mice received intraperitoneal SR144528 (n = 6, 0.1 mg/kg; n = 6, 1 mg/kg; n = 6, 2 mg/kg) for 3 months after confirmation of diabetes. An additional 6 diabetic mice received intraperitoneal cannabidiol (20 mg/kg) and 6 diabetic mice received intraperitoneal SR144528 (2 mg/kg) beginning at 4 months of age (3 months of diabetes) for one month for the comparison to intranasally delivered groups. Age- and sex-matched non-diabetic littermates were given either intranasal saline at equal volumes (n = 4) or intraperitoneal saline at equal volumes (n = 4) beginning at one week following STZ injections for 3 months, or after 3 months of diabetes (n = 4 each) for one month.

During Experiment 3, we sought to determine the impact of CB1 receptor ligation prior to and after establishment of a chronic neuropathic pain state. A total of 144 male CD1 wildtype diabetic mice and 16 male CD1 non-diabetic littermate wildtype mice were studied over the course of 4.5 months of diabetes (5.5 months of life). In diabetic mice, therapies were initiated at one week after STZ injections when diabetes was confirmed. A total of 18 received intranasal nabilone solution (non-selective CB1 and CB2 agonist) (Valeant Canada) (n = 6, 0.01 mg/kg; n = 6, 0.03 mg/kg; n = 6, 0.06 mg/kg) and a total of 18 diabetic mice received intranasal WIN55212-2 (selective CB1 agonist) (Sigma Aldrich Canada) (n = 6, 0.01 mg/kg; n = 6 0.03 mg/kg; n = 6, 0.06 mg/kg), both beginning at one week after STZ injection when diabetes was confirmed and continued for 3 months. WIN55212-2 has higher affinity than Δ^9^-Tetrahydrocannabinol for the CB_1 _receptor [40], but with a shorter half life. Eighteen diabetic mice received intranasal delivery of the selective CB1 cannabinoid antagonist SR141716A (GE Healthcare) (n = 6, 0.01 mg/kg; n = 6, 0.1 mg/kg; n = 6, 0.2 mg/kg) beginning at confirmation of diabetes and continued for 3 months. In order to examine the effect of CB1 receptor agonism in the presence of an established chronic neuropathic pain state, an additional 6 diabetic mice received intranasal WIN55212-2 (0.06 mg/kg), 6 mice received intranasal nabilone (0.06 mg/kg), and 6 diabetic mice received intranasal SR141716A (0.2 mg/kg) beginning at 4 months of age (3 months of diabetes) for one month. Intraperitoneal delivery was again performed for comparison of effect of intranasal delivery. Of the diabetic mice, a total of 18 received intraperitoneal nabilone (n = 6, 0.01 mg/kg; n = 6 0.3 mg/kg; n = 6, 0.6 mg/kg) while a total of 18 diabetic mice received intraperitoneal WIN55212-2 (selective CB1 agonist) (n = 6, 0.01 mg/kg; n = 6 0.3 mg/kg; n = 6, 0.6 mg/kg), each beginning after diabetes confirmation and continued for 3 months. Eighteen diabetic mice received intraperitoneal delivery of the selective CB1 cannabinoid antagonist SR141716A (n = 6, 0.1 mg/kg; n = 6, 1 mg/kg; n = 6, 2 mg/kg) for the 3 months beginning after diabetes confirmation. An additional 6 diabetic mice received intraperitoneal WIN55212-2 (0.6 mg/kg) and 6 diabetic mice received intraperitoneal SR141716A (2 mg/kg) beginning at 4 months of age (3 months of diabetes) for one month for the comparison to intranasally delivered groups. Age- and sex-matched non-diabetic littermates were given either intranasal saline at equal volumes (n = 4) or intraperitoneal saline at equal volumes (n = 4) beginning at one week following STZ injections for 3 months, or after 3 months of diabetes (n = 4 each) for one month.

In all cases, animals were inspected for nasal congestion, injection site infection, and for excessive sedation on a daily basis. Weight was monitored monthly to assess for cannabinoid-induced weight gain.

### Behavioural testing

In all cases, baseline testing was done immediately prior to STZ injections, and then immediately prior to cannabinoid agent administration. During maintenance therapy, behavioural testing was performed 12 hours after the daily administration was performed. A minimum of 1 hour was provided between forms of sensory testing, and a minimum of 5 mice underwent behavioural testing at each time point.

#### Mechanical Sensitivity

Mechanical withdrawal thresholds were tested using a Dynamic Plantar Aesthesiometer (Ugo-Basile, Milan). In brief, animals were placed in clear acrylic boxes (22 × 16.5 × 14 cm) with a metal grid floor in a temperature controlled room (22°C) and acclimatized for 15 min before testing. The stimulus was applied via a metal filament (0.5 mm) which applied a linearly increasing force ramp (2.5 g/s) to the plantar surface of the hind paw. A cut-off of 50 g was imposed to prevent any tissue damage. The force necessary to elicit a paw withdrawal was recorded. The paw withdrawal threshold (PWT) was calculated as the average of three consecutive tests with at least 5 min between each test. Mechanical allodynia was defined as reduced threshold after induction of diabetes compared to a baseline PWT before STZ injection.

#### Thermal Hyperalgesia

To quantitatively assess the thermal threshold of the hindpaw, mice were placed on the glass surface of a thermal testing apparatus with acclimatization for 30 minutes before testing. A mobile radiant heat source (Hargreaves apparatus) located under the glass was focused onto each of both hindpaws of each mouse. A radiant heat source was applied individually to the middle of either hindpaw for up to 60 seconds, with the latency (seconds) to withdrawal measured. Heating rate ramped from 30°C to 58°C over 60 seconds in consistent fashion on each occasion. The cutoff of 60 seconds was used to prevent potential tissue damage. Paws were inspected before and after thermal testing to ensure that no evidence of thermal damage was present. The withdrawal latency of both hindpaws from three consecutive trials was averaged, and the mean value was used as the thermal threshold. There were 5 minute intervals provided between trials.

### Electrophysiological testing

Electrophysiological assessment of sciatic nerve function was performed as previously described [38] under halothane anaesthesia. Initial baseline studies were carrier out prior to STZ or carrier injections. At least 4 mice in each intervention group underwent monthly electrophysiological testing beginning prior to induction of diabetes and after 1, 2, 4, 6, and 8 months of diabetes, as possible. For orthodromic sensory conduction studies, the sural nerve was used with a fixed distance of 30 mm from platinum subdermal stimulation needle electrodes (Grass Instruments, Astro-Med, West Warwick, RI) to the sciatic notch where recording electrodes were placed to measure the sensory nerve action potential (SNAP) amplitude and sensory nerve conduction velocity (SNCV). Near-nerve temperature was kept constant during testing at 37 ± 0.5°C using a heating lamp.

### Surgical harvesting

Following 1, 3, 4.5, 5 or 8 months of diabetes, as indicated, mice from each cohort were sacrificed using pentobarbital intraperitoneal injections. A total of 0.5 ml of whole intracardiac blood was obtained for HbA1C measurements to be performed with latex agglutination inhibition rate assay (Calgary Laboratory Services). The following tissues were harvested: dorsal lumbar spinal cord from L2-S1, and bilateral thalami. Left-sided tissues were placed in 2% Zamboni's fixative for later immunohistochemistry, while right-sided tissues were immediately fresh frozen at -80°C (Invitrogen, Burlington, ON) and stored at -80°C for later protein identification.

### Immunohistochemistry

After spinal cord and thalamus specimens were fixed in 2% Zamboni's fixative overnight at 4°C, they were washed in PBS, kept overnight in 25% glucose PBS solution, and then embedded in optimal cutting temperature embedding solution, before storage at -80°C until sectioning. Cryostat transverse and longitudinal nerve sections (10 μm) were placed onto poly-l-lysine and acetone-coated slides. Antigen retrieval was performed with slides placed in sodium citrate in an 80°C water bath, a PBS wash for 5 min, blocking with 10% goat serum for 1 h, and further PBS washing. In all cases, slides were incubated with primary antibody overnight at 4°C. After PBS washing, secondary fluorescent antibody was applied with incubation for 1 h at room temperature, followed by PBS washing and slide mounting.

Primary antibodies used were goat anti-ionized calcium-binding adaptor molecule 1 (Iba-1; 1:1000; Abcam, Cambridge, MA) for microglial identification, rabbit anti-cannabinoid receptor 1 antibody (CB1 receptor, 1:500, Sigma Aldrich Canada), rabbit anti-cannabinoid receptor 2 antibody (CB2 receptor, 1:500, Sigma Aldrich Canada), rabbit anti-phosphorylated p38 Mitogen Activated Protein Kinase (p-p38 MAPK; 1:100; Cell Signalling Technology, USA), and rabbit anti-microtubule associated protein-2 antibody produced in rabbit (MAP-2, 1:500, M3696, Sigma Aldrich Canada) for neuronal identification. Secondary antibodies used were either anti-rabbit IgG fluorescein isothiocyanate (FITC) labelled (1:100; Zymed, San Francisco, CA) or donkey anti-goat IgG CY3 labeled (1:200, Fitzgerald Industries, Concord, MA). Slides were cover-slipped with Vectashield mounting medium (Vector Laboratories, CA, USA) and visualised under a Zeiss Imager Z1 (Zeiss, UK) fluorescence microscope. Iba-1, p-p38 and GFAP-positive cells were determined by counting the number of profiles (cell bodies).

Slides were examined under fluorescence microscopy (Zeiss Axioskope, Axiovision and Axiocam, Zeiss Canada, Toronto, Canada) at 200×. Calculation of the number of immunofluorescent profiles as well as the relative luminosity was performed using Adobe Photoshop (Adobe Photoshop 9.0, Adobe, San Jose, CA, 2005). In both thalamic and dorsal spinal cord regions, the total numbers of microglia per transverse section, as well as the numbers of microglia with positive immunolabelling for markers of interest. In the ventral posterolateral nucleus (VPL) ventral posteromedial (VPM) and reticular (Rt) thalamic nuclei, microglia were identified using Iba-1 immunohistochemistry, while tertiary neurons were also identified using MAP-2 positivity along with study of positive immunolabelling for markers of interest. In the lumbar segment, the lateral, central and medial dorsal horn regions, representing laminae 1-3, were examined. When double immunolabelling was not possible within a single specimen due to antibodies deriving from identical species, consecutive slides were instead used and overlapped. Luminosity was classified as none-low (luminosity value of 0-150), moderate (150-250) or high (>250) using Adobe Photoshop software (scale of 0-255 with arbitrary units). For cellular densities, a box measuring 104 μm2 was placed onto areas of dorsal horn for regions between L2 and S1 and within thalamic nuclei of interest. A quantitative estimate (proportional area) of changes in the activation state of microglial cells was performed [41-43] in the VPL thalamic nucleus and dorsal spinal cord based on atlas boundaries and after subtraction of background signal. A resting microglia was classified as having a small, compact soma with long, thin, ramified processes. Activated microglia, in contrast, exhibit marked cellular hypertrophy, and retracted processes with process length less than soma diameter. Furthermore, microglia activation was assessed using a previously described qualitative scale: - for baseline staining; + for mild response; and ++ for moderate response [44]. All measurements were performed by a single examiner blinded to the group identity. A total of 25 randomly chosen areas of dorsal spinal cord and thalamus, from a minimum of 4 animals per cohort group were examined.

### Western Immunoblotting

Tissue portions from the dorsal spinal cord and thalamus were homogenized using a RotorStator Homogenizer in ice-cold lysis buffer (10% glycerol, 2% SDS, 25 mM Tris-HCl, pH7.4, Roche Mini-Complete Protease Inhibitors). Centrifugation of samples at 10,000G followed for 15 minutes and equal amounts (15 μg) of protein were separated by SDS-PAGE using 10% polyacrylamide gels under conditions previously described [38]. Blots were probed with Iba-1 (1:1000), CB1 (1:1000), CB2 (1:1000) and p-p38 MAPK (1:1000). For housekeeping, anti-β-actin (1:100, Biogenesis Ltd. Poole, UK) was applied to separate blots. Secondary anti-rabbit, anti-mouse, or anti-human IgG HRP Linked antibody (Cell Signaling) was applied at 1:5000 in each case as appropriate. Signal detection was performed by exposing of the blot to enhanced chemi-luminescent reagents (Amersham) for two minutes. The blots were subsequently exposed and captured on Kodac X-OMAT K film. In each case, three blots were performed from different composites of mice, and analyzed with Adobe Photoshop (Adobe Photoshop 9.0, Adobe, San Jose, CA, 2005) for semi-quantification of blotting density.

### Data analysis

Two-way repeated ANOVA measurements followed by Tukey's test were performed for analysis of behavioural studies. We chose to analyze behavioral data based upon comparison of either high or medium dosed intervention to low dose of the same intervention. We also chose to analyze behavioral data in mice with established diabetes and late intervention with comparison to diabetic mice who had received delivery of the same agent and with the same route in early intervention studies. One-way ANOVA followed by Tukey's test was used for immunohistochemistry. Data collected in the groups were expressed as mean ± standard error in all cases. For immunohistochemistry comparisons demonstrated as low/medium/high intensity, the individual values were compared using unmatched ANOVA testing.

## Results

### Experiment 1

#### Maintenance of Diabetes

After STZ injection, mice developed diabetes within 1 week in greater than 90% of animals, and in each case, diabetes was maintained over the length of the study. Diabetic mice were smaller than non-diabetic mice within 1 month after STZ injection and diabetic mice had smaller body weights throughout life (Table 1). Mouse glycated haemoglobin was increased in all diabetic mice after 9 months of life (Table 1). The mortality rate in diabetic mice was significantly higher than in non-diabetic mice (Table 1).

**Table 1:** Murine weights, fasting glycemia levels, glycated haemoglobin levels, and survival numbers in diabetic and non-diabetic mice.

Timepoint	Injection of STZ/Carrier	Month 1	Month 3	Month 5	Month 8
Murine Weight					

Non-Diabetic Mice	25.9 +/- 2.8 (n = 25)	33.8 +/- 3.8 (n = 20)	34.7 +/- 3.8 (n = 15)	41.9 +/- 3.9* (n = 10)	46.8 +/- 4.2* (n = 5)

Diabetic Mice	26.1 +/- 3.2 (n = 40)	26.9 +/- 4.2 (n = 33) (2 non-diabetic)	28.4 +/- 4.3 (n = 26)	30.6 +/- 5.7 (n = 18)	31.5 +/- 5.8 (n = 6)

Murine Glycemia and 8 Month HbA1C	Injection of STZ/Carrier	Month 1	Month 3	Month 5	Month 8 (Numbers in brackets indicate glycated haemoglobin levels)

Non-Diabetic Mice	5.4 +/- 2.2	5.7 +/- 2.6	6.0 +/- 2.6	6.1 +/- 3.2	6.4 +/- 3.1* *(5.4% +/- 1.8%)*

Diabetic Mice	5.5 +/- 2.0	32.1 +/- 5.3	32.5 +/- 5.1	32.4 +/- 5.8	32.6 +/- 5.4 *(14.7% +/- 2.1%)*^&^

Murine Survival Numbers	Injection of STZ/Carrier	Month 1	Month 3	Month 5	Month 8

Non-Diabetic Mice	25/25 (100%)	20/20 (100%)	15/15 (100%)	10/10* (100%)	5/5 (100%)*

Diabetic Mice	40/40 (100%)	33/33 (2 non-diabetic, 100%)	26/30 (87%)	18/25 (72%)	6/20 (30%)

All measures are mean +/- SEM for weights, glycemia levels, and haemoglobin A1C levels. * indicates significance at p < 0.05 with comparison of non-diabetic and diabetic mice cohort groups for weight and glycemic measures (non-matched ANOVA tests, *F*-values range between 5.68-137.9 for indicated groups and time points, DF ≥ 7,4, *n *≥ 5-10 at each time point as indicated. Glycated haemoglobin values are presented in italics in the 8 month column for glycemia levels. Note that 5 diabetic and non-diabetic mice were harvested each month as per protocol) or for survival (Kaplan-Meier Survival statistics, n values indicated).

#### Development of Diabetic Peripheral Neuropathy and Neuropathic Pain

**Figure 2:**
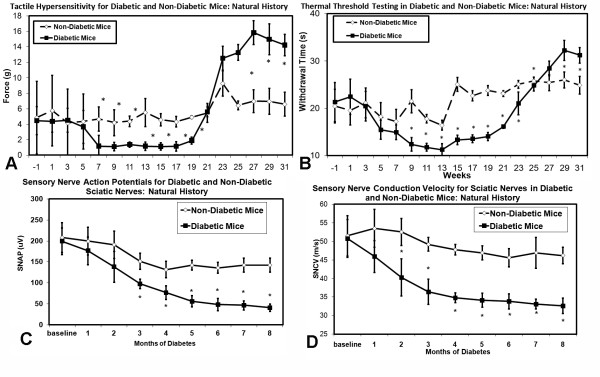
**Tactile (A) and thermal (B) sensory testing data mice with or without diabetes**. Significant differences were detected between the diabetic mouse and non-diabetic mouse groups (non-matched ANOVA tests, *F*-values range between 6.18-9.72 for indicated groups and time points, *n ≥ 5*, p < 0.05). Area under the curve (AUC) measurements were also significantly different between for the first 21 weeks of study (p < 0.05) [n = 5-10 mice in each mouse cohort for each time point]. Diabetic mice have a significant loss of sensory nerve action potential (SNAP) amplitudes (**C**) and sensory nerve conduction velocity (SNCV) (**D) **when compared to the non-diabetic mouse cohorts (non-matched ANOVA tests, *F*-values range between 2.13-5.88 for indicated groups and time points, *n ≥ 5*, p < 0.05) after 2-3 months of diabetes [n = 4-6 mice in each mouse cohort for each time point].

Diabetic mice developed tactile allodynia and thermal hyperalgesia at 7 and 9 weeks of diabetes respectively (Figure 2). After approximately 23 weeks of diabetes, hypersensitivity during these forms of testing disappeared in diabetic mice, and was followed by relative hyposensitivities. Electrophysiological testing demonstrated a loss of sensory nerve action potential amplitude and slowing of sensory nerve conduction velocity after 3 and 2 months of diabetes respectively (Figure 2).

#### Development of Increased Microglial Density and Activation

**Figure 3:**
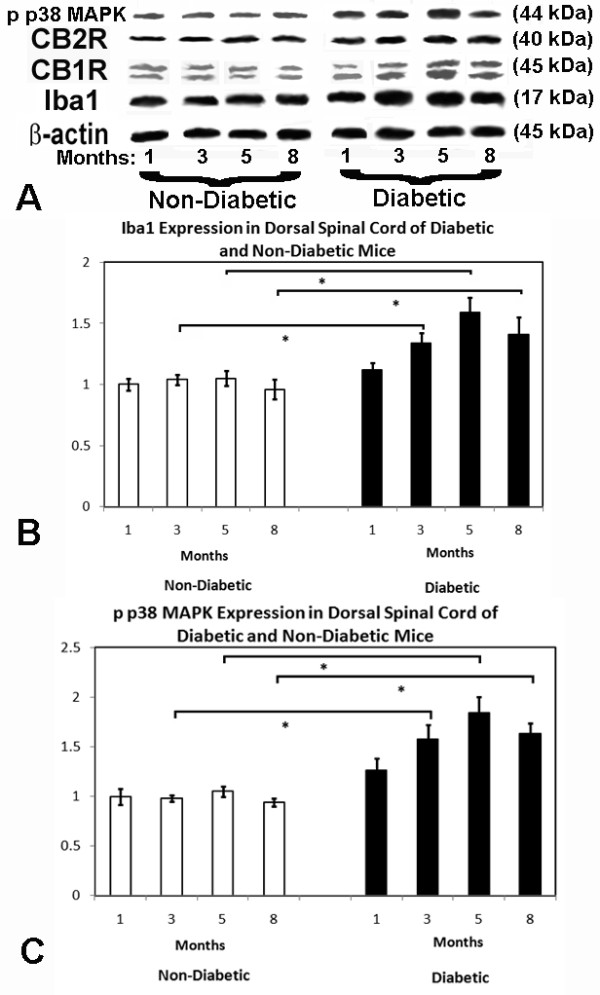
**During 8 months of diabetes, Western blotting (A) identified mild increasing levels of Iba1 (B), beginning at 3 months of diabetes, when compared to non-diabetic littermates**. As well, phosphorylated p38 MAPK elevates during diabetes until 5 months of time (**C**). Sample protein blots are demonstrated in **A **from a total of 3 sample blots identified for each marker and each time point. Multiple ANOVA tests were performed in each case, with * indicating significant difference (p < 0.05) between diabetic and non-diabetic cohorts.

Microglial quantification over the duration of diabetes identified increased microglial density in the dorsal spinal cord regions after 3 and 5 months of diabetes when compared to non-diabetic specimens (Table 2). Interestingly, there was a fall in microglial density after 8 months of diabetes, but diabetic specimens still maintained greater microglial quantities than non-diabetic specimens. In the thalamic regions, increased microglial density did occur after 5 months of diabetes, but was not identified at other time points; the extent of increased microglial density was considerably smaller than in the dorsal spinal cord regions. Iba-1 protein expression increased over time in diabetic spinal cord specimens (Figure 3) as compared to non-diabetic specimens. There was a non-significant trend towards increased levels of CB1/CB2 receptor expression in diabetic spinal cord specimens over time, while p-p38 MAPK expression elevated during the course of diabetes until the final time point when it declined (Figure 3). There were no increases in comparative protein levels within the diabetic thalamus (data not shown).

**Table 2:** Quantitative and qualitative analysis of microglia density within the dorsal horn and thalamus after indicated time points of diabetes

*Mouse Cohort*	Microglial Density in Ventral Lumbar Spinal Cord (number/mm^2^)			
	**1 Month**	**3 Months**	**5 Months**	**8 Months**

**Non-Diabetic**	167.3 ± 14.9 (-)	154.1 ± 11.3 (-)	142.1 ± 15.8 (-)	139.2 ± 13.4 (-)

**Diabetic**	183.8 ± 16.7 (-)	227.5 ± 14.2* (+)	308.4 ± 19.6* (+)	256.4 ± 22.5* (+)

	**Microglial Density in Thalamic Nuclei (number/mm^2^)**			

	**1 Month**	**3 Months**	**5 Months**	**8 Months**

**Non-Diabetic**	108.6 ± 10.2(-)	104.3 ± 9.8(-)	101.2 ± 9.6 (-)	99.5 ± 8.4 (-)

**Diabetic**	113.2 ± 12.4(-)	123.0 ± 11.4 (+/-)	128.3 ± 10.1* (+/-)	116.3 ± 13.7 (-/+)

All measures are mean +/- SEM for microglial densities. * indicates significance at p < 0.05 with comparison of non-diabetic and diabetic mice cohort groups (non-matched ANOVA tests, *F*-values range between 1.08-13.9 for indicated groups and time points, DF ≥ 7,4, *n *≥ 4 at each time point. Microglial activation is indicated using a qualitative scale: - for baseline staining; + for mild response; and ++ for moderate response, with the statistical mode response indicated in brackets, or where two values were similar, the greater response is shown in front of the second most common value.

We identified sufficient changes to indicate microglial activation in the diabetic dorsal spinal cord and lesser degrees of activation with the diabetic thalamus to proceed to further manipulation of the CB1/CB2 receptor system.

### Experiment 2

Diabetic mice were again smaller than non-diabetic mice within 1 month after STZ injection regardless of cannabinoid agent delivery. There was a non-significant trend towards larger weights in diabetic mice treated with intranasal or intraperitoneal cannabidiol. Mouse glycated haemoglobin levels remained increased in all diabetic mice after more than 9 months of life and were unchanged with cannabinoid agent delivery. Electrophysiological changes developed as witnessed in Experiment 1, without cannabinoid agents influencing the severity of electrophysiological changes developing with diabetes (data not shown). No systemic adverse effects were identified, and there was no excessive sedation detected to impact upon movement or food ingestion in any group.

**Figure 4:**
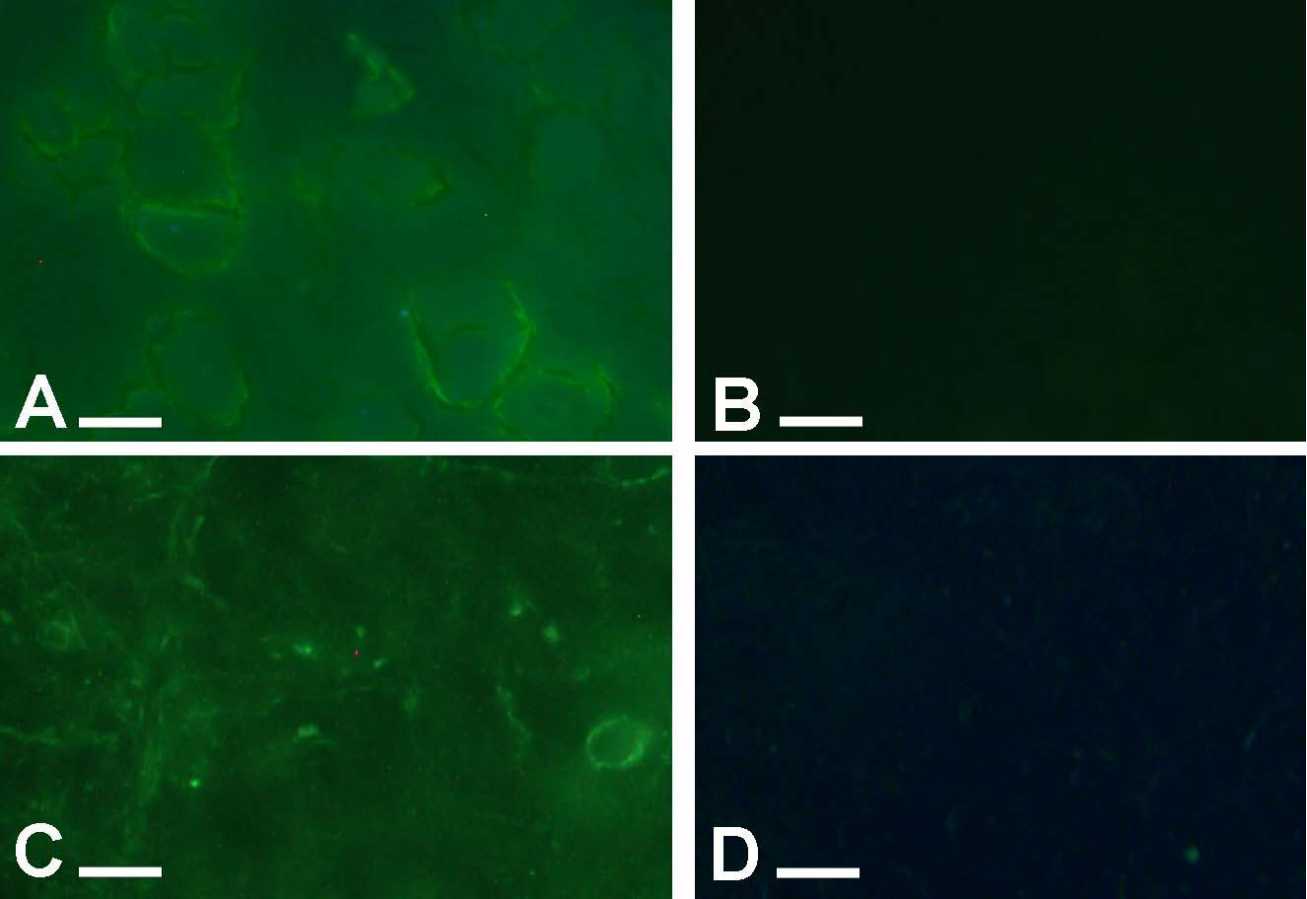
**Fluroescent labeled cannabidiol (A) was visible within diabetic mouse thalamus 6 hours after delivery, with saline intervention leading to no comparable fluorescence (B)**. Fluroescent labeled nabilone (C) was also visible within diabetic dorsal spinal cord 6 hours after delivery, with saline intervention leading to no comparable fluorescence (D). Bar = 10 μm

Fluorescently tagged cannabidiol was visualized in the thalamus after 1 and 6 hours and in the dorsal spinal cord after 6 and 24 hours (Figure 4) post-delivery when compared to saline delivery.

#### Assessment of Neuropathic Pain

**Figure 5:**
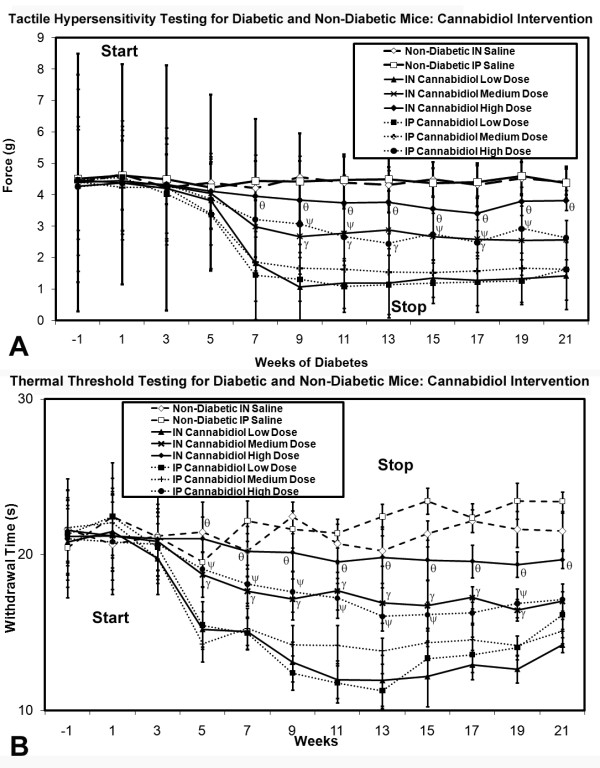
**Tactile (A) and thermal (B) sensory testing data for diabetic mice receiving either intranasal or intraperitoneal cannabidiol at low, medium, or high dose, with comparison to non-diabetic mice and saline delivery**. Diabetic mice receiving medium or high doses of intranasal or intraperitoneal cannabidiol had amelioration of both tactile allodynia and thermal hyperalgesia after 7 weeks when nociceptive behaviors began. This protection against the development of the neuropathic pain state was also noted continually after the stoppage of cannabidiol at week 14. For both tactile (**A**) and thermal (**B**) testing, significant differences were detected between the diabetic mouse group receiving medium (γ) or high doses (θ) of intranasal cannabidiol or high (Ψ) doses of intraperiteonal cannabidiol when compared to the diabetic mouse group receiving low dose intranasal or intraperitoneal cannabidiol respectively (non-matched ANOVA tests, *F*-values range between 0.88-4.13 for indicated groups and time points, *n ≥ 4*, p < 0.05). Area under the curve (AUC) measurements were also significantly different between the same comparison groups in each case (p < 0.05). [n = 4-10 mice in each mouse cohort for each time point]

**Figure 6:**
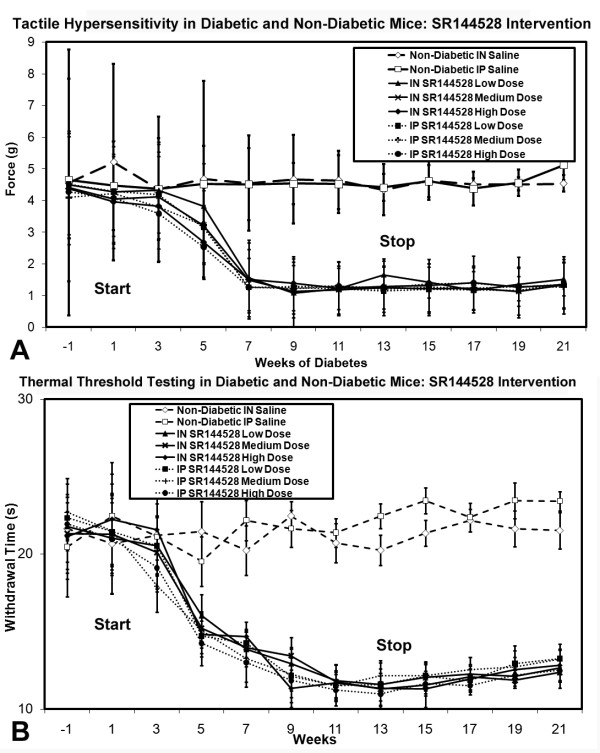
**Tactile (A) and thermal (B) sensory testing data for diabetic mice receiving either intranasal or intraperitoneal SR144528 at low, medium, or high dose, with comparison to non-diabetic mice and saline delivery**. There were no effects upon either tactile allodynia or thermal hyperalgesia development or maintenance in any group at any dose (non-matched ANOVA tests, p NS). [n = 4-10 mice in each mouse cohort for each time point].

**Figure 7:**
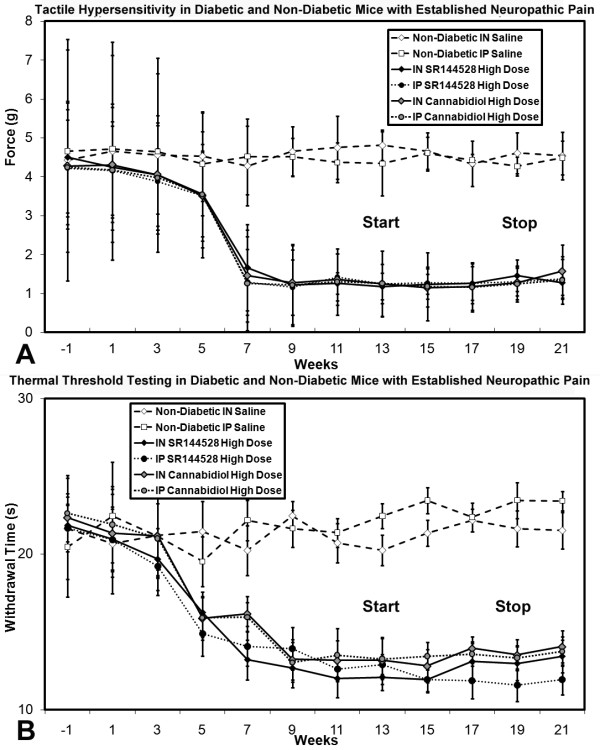
**Tactile (A) and thermal (B) sensory testing data for mice with established diabetes receiving either intranasal or intraperitoneal cannabidiol or SR144528 at low high dose, with comparison to non-diabetic mice receiving intranasal or intraperitoneal saline**. There were no effects upon either tactile allodynia or thermal hyperalgesia development or maintenance in any group at any dose (non-matched ANOVA tests, p NS). [n = 4-10 mice in each mouse cohort for each time point] despite early intervention studies for cannabidiol demonstrating protection against development and maintenance of neuropathic pain.

Diabetic mice receiving intranasal cannabidiol at moderate or high doses beginning at onset of diabetes or receiving intraperitoneal high dosing were subject to alleviation of development of thermal hypersensitivity and tactile allodynia. This effect was maintained during cannabidiol delivery and for the additional four assessments over 2 months following discontinuation of cannabidiol (Figure 5). Meanwhile, diabetic mice receiving intranasal or intraperitoneal SR144528 at the onset of diabetes had no measureable effect upon the development of either thermal hypersensitivity or tactile allodynia (Figure 6). In mice with established neuropathic pain due to diabetic peripheral neuropathy, neither intranasal/intraperitoneal cannabidiol nor SR144528 had any effect upon already present and chronic thermal hypersensitivity or tactile allodynia (Figure 7).

#### Development of Increased Microglial Density and Activation

**Figure 8:**
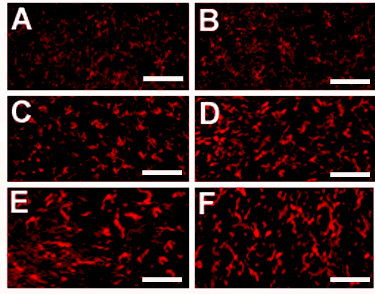
**After several months of diabetes, microglia accumulation can be noted within dorsal spinal cord and to a lesser degree within thalamic nuclei**. Compared to non-diabetic age-matched mice (**A**), immunohistochemistry identified a mild but significant accumulation of microglia within the thalamus after 5 months of diabetes (**B**). Microglial accumulation and activation (evaluated qualitatively) was more substantial in the dorsal spinal cord of diabetic mice. Treatment with intranasal high dose cannabidiol (**C**) attenuated the microglial accumulation identified in age-matched diabetic mice reciving no intervention (**D**) or receiving intranasal (**E**) or intraperitoneal (**F**) WIN55212-2 (see Tables 2 and 3). Bar = 50 μm

Those mice receiving medium or high doses of intranasal/intraperitoneal cannabidiol demonstrated lower densities of microglia in the dorsal spinal cord (Table 3, Figure 8) at endpoint. Expression of p-p38 MAPK was also diminished in these mice. Diabetic mice receiving intranasal/intraperitoneal SR144528 had no detectable changes in microglia density or p-p38 MAPK expression. Qualitative assessment of microglial activation revealed no definite differences between cohort groups. Western blotting confirmed lower levels of both Iba-1 and p-p38 MAPK protein expression in the dorsal spinal cord (Figure 8) of mice who received cannabidiol at diabetes onset. There were no changes in microglial density or protein expression identified with interventions for thalamic tissue specimens. There were also no observed changes in microglial density or protein expression when late interventions were provided to mice with long standing diabetes.

**Table 3:** Quantitative and qualitative analysis of microglia density within the dorsal horn and thalamus with cannabinoid interventions

*Mouse Cohort*	Microglial Density (number/mm^2^)	
	**Ventral Lumbar Spinal Cord**	**Thalamic Nuclei**

**Non-Diabetic**		

*Intraperitoneal*		

**Saline**	147.3 ± 17.2 (-)	99.2 ± 9.9 (-)

*Intranasal*		

**Saline**	136.1 ± 19.2 (-)	105.3 ± 9.3 (-)

**Diabetic**		

*Intraperitoneal*		

Early Intervention		

Cannabidiol	228.6 ± 15.3* (+)	116.5 ± 9.4 (+/-)

SR144528	316.6 ± 17.8 (+)	127.5 ± 11.3 (+/-)

Nabilone	309.3 ± 18.2 (+)	125.6 ± 10.5 (+/-)

WIN55212-2	305.7 ± 20.5 (+)	124.4 ± 9.7 (+/-)

SR141716A	314.7 ± 16.9 (+)	130.0 ± 10.4 (+/-)

Late Intervention		

Cannabidiol	314.2 ± 18.8 (+)	118.2 ± 10.8 (+/-)

SR144528	312.5 ± 19.4 (+)	125.6 ± 9.5 (+/-)

Nabilone	319.5 ± 19.9 (+)	124.5 ± 9.2 (+/-)

WIN55212-2	311.3 ± 18.9 (+)	131.2 ± 11.2 (+/-)

SR141716A	325.3 ± 17.7 (+)	129.0 ± 9.0 (+/-)

*Intranasal*		

Early Intervention		

Cannabidiol	209.5 ± 14.8* (+)	114.7 ± 12.2 (+/-)

SR144528	302.3 ± 18.2 (+)	125.4 ± 11.3 (+/-)

Nabilone	301.1 ± 16.5 (+)	132.5 ± 11.7 (+/-)

WIN55212-2	319.1 ± 18.4 (+)	126.1 ± 8.9 (+/-)

SR141716A	316.3 ± 17.8 (+)	130.5 ± 9.6 (+/-)

Late Intervention		

Cannabidiol	314.5 ± 18.1 (+)	120.0 ± 9.5 (+/-)

SR144528	322.6 ± 20.5 (+)	133.4 ± 11.6 (+/-)

Nabilone	328.1 ± 19.9 (+)	125.6 ± 11.1 (+/-)

WIN55212-2	321.1 ± 18.0 (+)	122.0 ± 9.0 (+/-)

SR141716A	312.6 ± 18.4 (+)	124.6 ± 9.4 (+/-)

All measures are mean +/- SEM for microglial densities. * indicates significance at p < 0.05 with comparison of diabetic mice cohort groups receiving high and low dose cannabidiol through either intranasal or intraperitoneal routes (non-matched ANOVA tests, *F*-values range between 1.89-3.92 for indicated groups and time points, DF ≥ 7,4, *n *≥ 4 at each time point. Microglial activation is indicated using a qualitative scale: - for baseline staining; + for mild response; and ++ for moderate response, with the statistical mode response indicated in brackets, or where two values were similar, the greater response is shown in front of the second most common value.

### Experiment 3

As in Experiment 2, diabetic mice were smaller than non-diabetic mice, and had characteristic electrophysiological changes and elevated HbA1C at endpoint; these measures were unchanged with cannabinoid agent delivery (data not shown).

Fluorescently tagged nabilone was also visualized in the thalamus after 1 and 6 hours and in the dorsal spinal cord after 6 and 24 hours (Figure 4) post-delivery when compared to saline delivery.

#### Assessment of Neuropathic Pain

**Figure 9:**
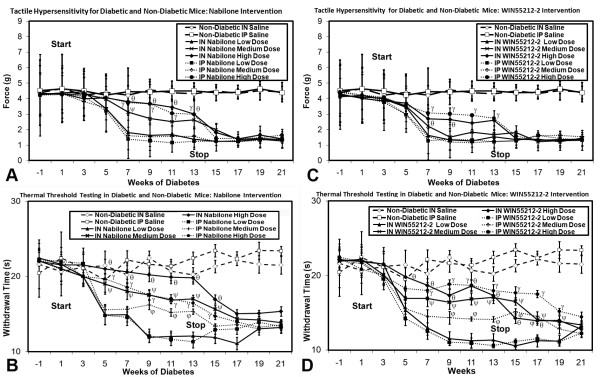
**Tactile (A) and thermal (B) sensory testing data for diabetic mice receiving either intranasal or intraperitoneal nabilone at low, medium, or high dose, with comparison to non-diabetic mice and saline delivery is shown**. Those diabetic mice receiving medium or high doses of intranasal or intraperitoneal nabilone had amelioration of both tactile allodynia and thermal hyperalgesia beginning at week 7 when nociceptive behaviors began. This protection against the development of the neuropathic pain state disappeared after discontinuation of nabilone at week 14. Tactile (**C**) and thermal (**D**) sensory testing data for mice with diabetes receiving either intranasal or intraperitoneal WIN55212-2 at low, medium, or high dose, with comparison to non-diabetic mice receiving intranasal or intraperitoneal saline is also demonstrated. Those diabetic mice receiving high doses of intranasal or intraperitoneal WIN55212-2 had amelioration of tactile allodynia beginning at week 7 when nociceptive behaviors began, while thermal hyperalgesia was modulated by either medium or high doses of intranasal or intraperitoneal WIN55212-2. This protection against the development of the neuropathic pain state disappeared after discontinuation of nabilone at week 14, after which all diabetic mouse cohorts had comparable levels of tactile allodynia and thermal hyperalgesia. For both tactile (**A, C**) and thermal (**B, D**) testing, significant differences were detected between the diabetic mouse group receiving medium (γ) or high doses (θ) of intranasal nabilone/WIN55212-2 or medium (φ) high (Ψ) doses of intraperiteonal nabilone/WIN55212-2 when compared to the diabetic mouse group receiving low dose intranasal or intraperitoneal nabilone/WIN55212-2 respectively (non-matched ANOVA tests, *F*-values range between 0.76-3.07 for indicated groups and time points, *n ≥ 4*, p < 0.05). Area under the curve (AUC) measurements were also significantly different in each case (p < 0.05) for weeks 1-13, but not for assessment of tactile allodynia in mice receiving any dose/route for WIN55212-2 (**C**). [n = 4-10 mice in each mouse cohort for each time point]

Diabetic mice receiving intranasal/intraperitoneal nabilone or WIN55212-2 solution in moderate-high doses at onset of diabetes demonstrated alleviation of both tactile allodynia and thermal hyperalgesia (Figure 9). This dose-dependent benefit was only observed during the weeks of nabilone or WIN55212-2 delivery, and tactile allodynia and thermal hyperalgesia of greater magnitude developed in the weeks following discontinuation of nabilone. In contrast, intranasal/intraperitoneal SR141716A delivery failed to impact upon the development of tactile allodynia or thermal hyperalgesia in any amount (data not shown). No systemic adverse effects were identified, and no excessive sedation was noted to impact upon movement or food ingestion.

**Figure 10:**
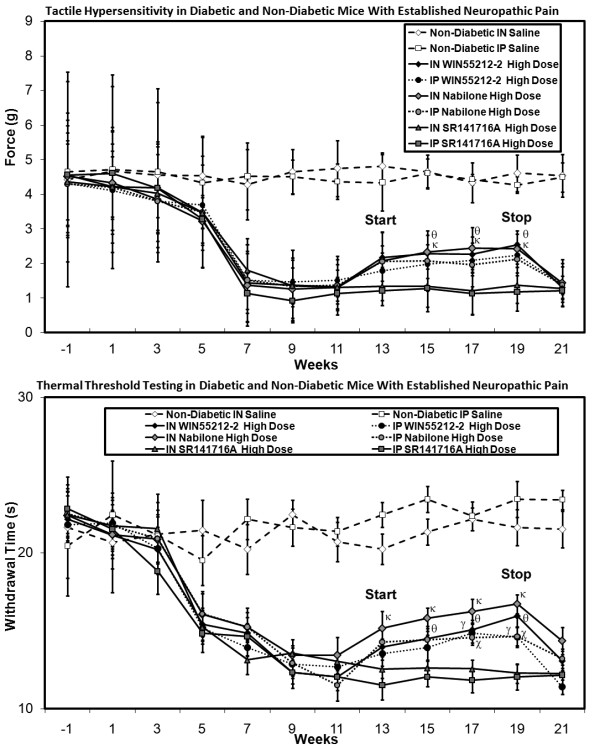
**Tactile (A) and thermal (B) sensory testing data for mice with established diabetes receiving either intranasal or intraperitoneal cannabinoid agents, with comparison to non-diabetic mice and saline delivery**. Diabetic mice receiving medium and high doses of intranasal nabilone or WIN55212-2 had improvement of thermal hyperalgesia +/- tactile allodynia beginning at week 13 when interventions began. Stoppage of cannabinoid agents saw resumption of the full neuropathic pain state at week 21. For both tactile (**A**) and thermal (**B**) testing, significant differences were detected between the diabetic mouse group receiving moderate (γ) or high doses (θ) of intranasal cannabidiol or medium (χ) or high (τ) doses of intraperiteonal cannabidiol when compared to the diabetic mouse group receiving low dose intranasal or intraperitoneal cannabidiol at outset respectively (non-matched ANOVA tests, *F*-values range between 0.95-2.85 for indicated groups and time points, *n ≥ 4*, p < 0.05). Area under the curve (AUC) measurements were also significantly different between the same comparison groups in each case (p < 0.05). [n = 4-10 mice in each mouse cohort for each time point]

In mice with chronic diabetic peripheral neuropathy and established neuropathic pain, high dose intranasal/intraperitoneal nabilone or WIN55212-2 partially alleviated existing tactile allodynia and thermal hyperalgesia, but there was no observed effect with any intervention using SR141716A (Figure 10).

#### Development of Increased Microglial Density and Activation

**Figure 11:**
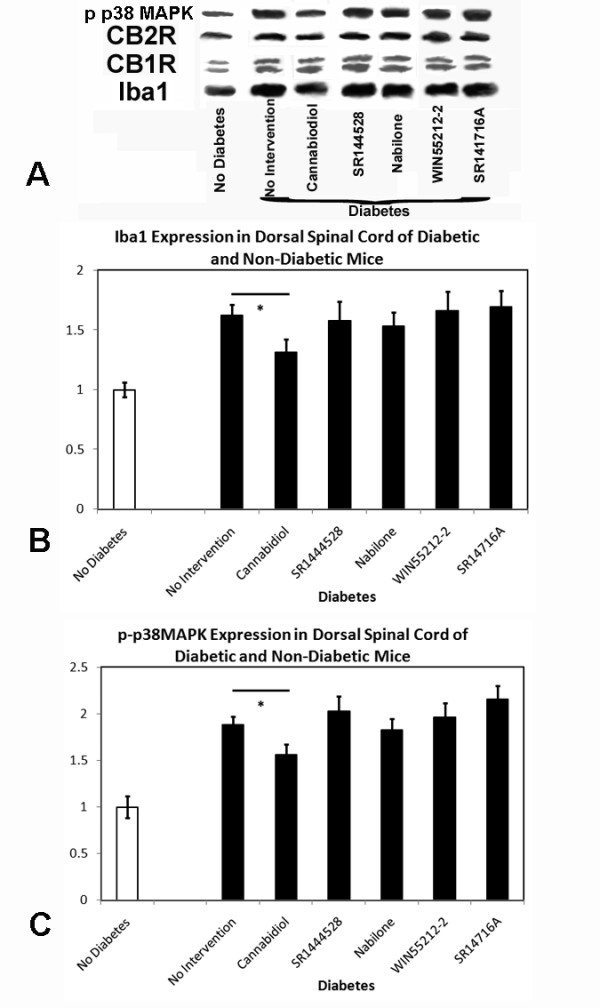
**During 5 months of diabetes, Western blotting (A) identified mild increased levels of Iba1 (B) in diabetic mice**. As well, phosphorylated p38 MAPK was elevated during diabetes at 5 months of time (**C**). Sample protein blots are demonstrated in **A **(total of 3 sample blots identified for each marker and each intervention at the final time point). Only intranasal or intraperitoneal cannabidiol administered at the onset of diabetes was associated with suppression of Iba1 and phosphorylated p38 MAPK levels at endpoint (**B, C**). Multiple ANOVA tests were performed in each case, with * indicating significant difference (p < 0.05) between diabetic cohorts receiving intranasal cannabidiol and no intervention after 5 months of diabetes.

Despite transient improvement in neuropathic pain measures, diabetic mice receiving intranasal/intraperitoneal nabilone, WIN55212-2, or SR141716A had no differences noted for microglial densities (Table 3) and no visible changes in protein expression of Iba-1 or p-p38 MAPK within the dorsal spinal cord (Figure 11). There were no identified changes in microglial density or protein expression for mice receiving interventions within thalamic tissue specimens. There were also no observed changes in microglial density or protein expression when late interventions were provided to mice affected by long standing diabetes.

## Discussion

STZ-induced diabetes and subsequent tactile allodynia/thermal hypersensitivity can be ameliorated with CB1 and CB2 receptor agonists. In addition, the CB2 agonist cannabidiol provided at the onset of diabetes and prior to identified neuropathic pain limited the development of later neuropathic pain and prevented increases in microglial density. These results verified our hypothesis that CB2 receptor ligation prevents microglial accumulation after development of a neuropathic pain state, although we did not identify any obvious differences in qualitative assessment of microglial activation nor differences in electrophysiological detection of diabetic neuropathy. Along with smaller numbers of accumulated microglia, we observed an increase in the phosphorylated form of p38 MAPK which was diminished in diabetic mice receiving CB2 receptor agonist, suggesting a more limited state of microglial activation. Meanwhile, provision of higher doses of CB1 agonists were effective in relieving tactile allodynia/thermal hypersensitivity, but failed to impact upon later neuropathic pain once discontinued and failed to impact upon microglial numbers. Therefore, the CB1 agonists transiently benefitted a developing or pre-existing neuropathic pain state in this model of diabetic peripheral neuropathy. It was anticipated that nabilone, a CB1 and CB2 receptor agonist may impact upon the development of neuropathic pain, but it was only associated with transient improvement in neuropathic pain parameters.

### Microglial Activation

Microglia, functionally equivalent to peripheral macrophages, entangle with central neurons and play a key role in pathophysiological pain [45]. Activation of spinal microglia can be observed in a number of neuropathic pain models following peripheral nerve injury [5,7]. It has been demonstrated that intrathecal delivery of minocycline or systemic propentofylline, compounds inhibiting microglia activation, reduces the development of induced sensory hypersensitivity in post-traumatic nerve injury models [46,47]. CB2 agonists also appear to limit microglia accumulation and activation in a murine chronic neuropathic pain state.

The signal by which nerve injury or disease may lead to microglial activation is unclear, but may relate to acute release of ERK, an enzyme that plays a critical role in intracellular signal transduction and neuronal plasticity. When ERK activates within damaged neurons, it may set into motion a series of neuronal changes that lay the foundations for altered sensory processing [48]. In the hours following an acute injury, ERK becomes activated in surrounding microglia where it remains active for days and weeks, upholding microglial support of the pain phenotype. Later, activation within astrocytes may also occur. This pattern of ERK activation following nerve injury likely influences ionotropic channel redistribution along the damaged neuron and its neurites, perhaps contributing to hypersensitive behaviors [48]. An example of this is the increased expression of Na_v_1.3 channels within second-order spinal cord dorsal horn neurons and third-order thalamic neurons after traumatic nerve injuries; these likely contribute to amplification of hyperexcitability and unfortunate maintenance of the pain state [16]. In addition, integrative functions, such as signal gating [49,50], within the dorsal horn modulate sensory signals on their travels from the peripheral nervous system to the brain. Modifications in ERK and channel function may lead to in appropriate amplification of signals, contributing to central sensitization. Another marker of microglial activation, increased phosphorylation of the p38 MAPK [14] was also observed in spinal microglia following development of diabetes during a chronic neuropathic pain state. Previous shorter duration studies of diabetes in rats [3] were not able to demonstrate change in the levels of p38 MAPK phosphorylation in the spinal cords [15]. Such changes in p38 MAPK phosphorylation seem to be limited to microglia, [51] which demonstrate substantial increased expression, and occur across many painful conditions[52].

### Microglial Activation in Diabetes

In addition to acute traumatic injury models, diabetic mice also exhibit enhanced microglial cell densities in the dorsal horn of the spinal cord, accompanied by some mild qualititative morphological changes, felt to be related to diabetic peripheral neuropathy pain. Morphological changes similar to ours reported here have been demonstrated in four week STZ-induced diabetic rats [15]. In our model, there is no acute or sudden injury to the nervous system as with a traumatic nerve injury model, so diabetes seemed to lead to a more gradual rise in microglia and p-p38 MAPK elevation than seen in other models. It appears as though with progression of the disease, enhanced p38 phosphorylation occurs within months, but then may wane in the late time points of our murine diabetic model. This rise and fall of p-p38 MAPK expression also parallels microglial density fluctuations. Although more easily detected in acute injury models, this greater duration of time to achieve microglial density increases and activation may also provide a greater window for therapeutic management. It may also be possible to modulate p38 MAPK to contribute to antinociceptive effects [14,46], although this may also affect glycemic levels, confounding the purported mechanism by which p38 MAPK may affect nociception.

The diabetic mouse model used showed latency to onset of neuropathic pain features and late resolution of allodynia and hyperpathia with replacement by anesthesia. Although this latency in NeP onset may be due to gradual accumulation and activation of microglia, other mechanisms are likely contributory as well. Central sensitization with activity-dependent changes in synaptic activity at the dorsal horn and other central nervous system structures likely develop [53]. Ectopic activity occurring due to excessive activity in voltage gated calcium and sodium channels also contributes to sensitization [54,55]. Time-dependent trafficking of receptors or ion channels may also have delayed onset with prolonged effects. After prolonged diabetes, human DPN patients may have remission of associated NeP [56] similar to the phenomenon seen in our model - this may be due to the more complete loss of intraepidermal nerve fibers at this time witnessed in murine models of DPN [38]. Overall, the STZ mouse model used provides a good approximation of human diabetic neuropathy, justifying its study for pathogenesis of NeP.

### Cannabinoids and Their Receptors

Cannabinoids have a diverse range of effects on the central nervous system, mediated by the activation of the CB receptors coupled to G-proteins ligated by endogenous ligands, the endocannabinoids. The endocannabinoids have a unique mode of action, with retrograde synaptic inhibition of the release of neurotransmitters such as glutamate [57]. CB2 receptors are associated with immune cells, microglia and have been identified in the peripheral nervous system. CB2 agonists successfully modulate inflammatory responses [58]. Both CB1 and CB2 receptors are expressed in activated microglial cells, but their cellular localization differs. Modifications in the cellular localization of CB receptors are associated with a change in their functionality, particularly with CB2 receptors [59], suggesting that functionality is regulated by plasmalemma translocation. Such changes may impact upon functions such as migration, as CB2 receptors abundantly accumulate at the leading edges of activated microglia [26]. There is certainly an inflammatory component in neuropathic pain models, although it is not yet understood if ongoing inflammation or inflammatory mediators maintain chronic neuropathic pain. The microglial accumulation in the dorsal spinal cord in our experiments indicates that such changes also occur in prolonged diabetes. After 8 months of diabetes in our murine model, microglial numbers began to fall, so neuroinflammation in a chronic pain model may peak before waning after a lengthy duration. The pro-inflammatory actions of glia are involved in neuropathic hypersensitivity [7], and the anti-inflammatory effects of CB2 agonists had a central role in our study in the prevention of neuropathic pain development.

There is a large body of evidence that cannabinoids are effective analgesics in pain, including forms of acute pain, inflammatory pain and neuropathic pain [60]. Previous studies with CB2 agonists have demonstrated development of anti-nociception [61]. Selective CB2 agonists modulate tactile allodynia in nerve injured rats and mice [62], and a CB2 agonist was also of benefit in our model of chronic diabetes and diabetic peripheral neuropathy. CB2-mediated antinociception also occurs in inflammatory hyperalgesia and acute nociception [61,63,64] as well as in more chronic diseases such as with experimental diabetes [65]. The mechanism by which CB2 receptor agonists modulate neuropathic pain is uncertain, but is likely to be related to inhibition of inflammatory cell activity. Neuropathic pain requires ongoing sensitization due to a constant afferent bombardment emenating from the injured nervous system [66] or changes in the dorsal root ganglion such as with sympathetic sprouting [67]. Cannabinoids may suppress such ectopic input and alter central sensitization by modifying inflammatory activity at the site of nervous system injury. It is entirely possible that other CB2-mediated mechanisms exist as well. In our study, it was only the selective CB2 agonist which led to diminished microglial densities and p-p38 MAPK expression within the dorsal spinal cord, associated with at least partial protection against development of neuropathic pain even after the CB2 agonist was withdrawn. This effect can be antagonized by concurrent CB2 antagonists [62] or selective CB1 antagonists [68] in some studies. It was somewhat unanticipated in our results that the CB2 antagonist and the CB1 antagonist were not pronociceptive; similar results have been demonstrated in traumatic nerve injury models of neuropathic pain [69]. This lack of effect may have been related to insufficient dosing or choice of delivery routes. The lack of effect of both CB1 and CB2 antagonists may also relate to persistant tonic activity in the endogenous cannabinoid system [60].

### Intranasal Delivery for Painful States

Although intranasal delivery has been initiated for potential therapy of cognitive dysfunction, ischemic disease, or demyelinating diseasethe use of intranasal agent delivery for pain management has been limited to the use of fentanyl [70] and ketamine [71]. These results demonstrate that in mice, intranasal delivery of cannabinoid agents is at least as effective as intraperitoneal delivery. Future human studies may assist in the further assessment of the roles of cannabinoids in chronic pain prevention and alleviation.

### Limitations of the Present Study

There are limitations to the present study that require discussion. It is possible that the response to different acute or chronic nociceptive stimuli such as tactile and thermal stimuli may not involve cannabinoid-sensitive peripheral components. Our investigations were limited to specific agonists and antagonists at three doses, but other cannabinoid agonists/antagonists and doses may lead to different behavioural and inflammatory results than presented here. Concentrations selected for use were based upon prior publications and may not have been most optimal for calculation of intranasal dosing, although our selected doses were associated with definite behavioural changes. Although we describe diabetic peripheral neuropathy pain as the main stimulus for migroglial density increases, it is conceivable that diabetes itself could lead to the changes.

## Conclusions

The present data confirm the efficacy of cannabinoid agonists, both for the CB1 and CB2 receptor, in modulation of acute thermal and tactile hypersensitivity as features of neuropathic pain. Furthermore, CB1 agonism from the onset of the offending stimulus (diabetes) normally leading to neuropathic pain ameliorated the development of a neuropathic pain state. In contrast, we did not demonstrate worsening of the neuropathic pain state with CB1 or CB2 antagonists. Overall, this suggests a greater role for CB2 receptors in neuropathic pain, including a chronic diabetic peripheral neuropathy state, than has previously been described. Such selective targeting with either CB2 selective agonists may play a role in the prevention of neuropathic pain states if treatment could be delivered at the time of neural injury or disease.

## Abbreviations

2-AG: 2-arachidonoylglycerol; ANOVA: Analysis of variance; CB: Cannabinoid receptor; CB1: Cannabinoid receptor-1; CB2:Cannabinoid receptor-2; CNS:Central nervous system; DPN: Diabetic peripheral neuropathy; FITC: Fluorescein isothiocyanate; HbA1C: Glycated haemoglobin A1C; Iba-1: ionized calcium binding adaptor molecule *1*; IL-1β: Interleukin-1beta; IL-6:Interleukin-6; MAP-2: Microtubule associated protein-2; MAPK: Mitogen-activated protein kinase; NeP: Neuropathic pain; PBS:Phosphate Buffered Solution; PNS: Peripheral nervous system; Rt: Reticular thalamic nucleus; SDS-PAGE:Sodium dodecyl sulfate polyacrylamide gel electrophoresis; SNAP:Sensory nerve action potential; SNCV: Sensory nerve conduction velocity; STZ: Streptozotocin; TNFα: Tumor necrosis factor-alpha; THC:Tetrahydrocannabinol; VPL: Ventral posterolateral nucleus; VPM: Ventral posteromedial nucleus.

## Competing interests

The authors declare that they have no competing interests.

## Authors' contributions

CT conceived of the study, participated in its design and coordination and drafted the manuscript carried out. CT also performed the surgeries and molecular studies related to the project. NP and CE carried out the delivery of agents and performed behavioral testing in all cases, and assisted with surgeries. WF participated in study design and helped to draft the manuscript. All authors read and approved the final manuscript.
